# A Visualized and Scientometric Analysis of Health Literacy Research

**DOI:** 10.3389/fpubh.2021.811707

**Published:** 2022-01-25

**Authors:** Jian Wang, Fakhar Shahzad

**Affiliations:** ^1^College of Economics and Management, Zhengzhou University of Light Industry, Zhengzhou, China; ^2^School of Management, Jiangsu University, Zhenjiang, China

**Keywords:** health literacy, visualized analysis, Scientometric analysis, Citespace, healthcare

## Abstract

**Purpose:**

The health literacy concerned by numerous policy and advocacy organizations is springing up rapidly and has evolved substantially over the past few decades. During the period of COVID-19, people who are in the absence of effective treatment and limited availability of vaccination need a higher health literacy to protective themselves. In order to provide a summary of the health literacy research, a visualized and Scientometric analysis is applied in this study.

**Methods:**

Based on a scientific review of 3,670 data on health literacy from Web of Science, this research uses Citespace software to systematically and objectively describe health literacy to determine the knowledge evolution structure between articles and investigate research trends.

**Results:**

The results show that the annual outputs of publications have grown rapidly since 2003. The USA and Australia make larger contributions compared with other countries in terms of quantity of publications and worldwide collaboration relationship between them. The “Soc Sci Med,” “J Health Commun.” “Patient Educ Couns” and “J Gen Intern Med” pay more attention to health literacy research. The leading authors with influence and authority are Wolf MS, Osborne RH, and Paasche-Orlow MK. Health literacy research in this field focuses on care, knowledge, and education, and so on. An emerging trend of health literacy with Covid-19, online tools, nursing, and obesity will spread across the globe.

**Conclusion:**

Compared with simply reviewing existing articles, the major contribution in this study is a comprehensive review of yearly scientific output, journals, countries, institutions, contributors, highly cited papers, and keywords for health literacy research. The review also provides valuable and seminal guidelines for interested researchers on health literacy research.

## Introduction

Along with changing demographics, significant advancement in science and technology, improvement of individuals' health beliefs as well as the complex demands of health in modern society, individuals and counties pay more attention to the health knowledge and skill to maintain or improve quality of life. Many countries or organizations, such as the EU (1, 2), American (3), and Australia (4, 5) have enacted policies to promote health literacy. Amid the COVID-19 pandemic, dealing with an overabundance of information rife with misinformation and hoaxes requires adequate health literacy to improve public health worldwide (6). However, there are still challenges with low health literacy to embed health literacy principles into effective self-management of diseases. On these grounds, health literacy is a crucial factor associated with health care and has greatly attracted scholars' interest. Many definitions for health literacy have been proposed from different perspectives. For example, health literacy is the skill or ability to retrieve, understand, analyze and act on this information as defined by the American Medical Association Foundation (7). Nutbeam divided the evolving concept of health literacy into two different views, respectively, as a clinical “risk,” or a personal “asset” (8). Based on the above definitions, several relevant studies on health literacy from different disciplines have been carried out to enhance their ability to recognize disorders and seek effective treatments (6, 9–18).

However, it is difficult for researchers to understand the current status and development trends from numerous qualitative and quantitative researches from a single perspective. For example, some studies concentrated on the assessment of health literacy (19–21). Some scholars investigate the relationship between health literacy and health behavior (6, 12, 15, 16, 22). In a word, a comprehensive, structured overview of health literacy is of great necessity. The literature reviews for a specific topic of health literacy are performed in terms of region difference [e.g. Malaysia (23), China (24), and Singapore (25)], age difference [e.g., young adults (26), older adults (27), and children (28)] or gender difference [e.g., women (24), men (29)] and so on. An exploratory bibliometric analysis of health literacy covering the period 1997–2007 is conducted by Bankson, but only including analyzing the characteristics of literature amount and core journals (30). Therefore, it is necessary to summarize previous research results and update the emerging trend and hotspots in health literacy research.

Given the incomplete and out-of-date literature research, this paper will use Scientometric and visualized analysis to demonstrate knowledge structures and developments in the field of health literacy from 2003 to 2021 with the help of Citespace.

The manuscript is organized as follows. In the next section, this study describes the materials and methods used to acquire the bibliometric analysis data. The following section contains the detailed results involving visualization and analysis on the distribution of scientific output, main source journals, countries, institutions, contributors, highly cited articles, and keywords. The final section includes the main findings, limitations, and future research.

## Materials and Methods

### Material Collection

The literature data in the areas of health literacy collected from the SCIE and SSCI citation index database in the Web of Science Core Collection are cover more scientific, comprehensive, accessible, and authoritative journals papers than other databases. Therefore, the data used in this study come from the index of Science Citation Index Expanded (SCI-EXPANDED), Social Sciences Citation Index (SSCI) of WOS Core Collection databases. A rapid upward growth pattern of published articles on health literacy after 2003 can be explained by the publicities undertaken by the Institute of Medicine, the American Medical Association, and the Medical Library Association (MLA) around this time (30, 31). Therefore, this study downloaded health literacy-related multidisciplinary studies since 2003. The time period of these publications is from 2003 to 2021 for an integral vision of the research development status and the evolution experienced during the last two decades by the research field. In the final data set, document type only retains the journal paper. Based on the retrieval strategy described above, data containing the terms “health literacy” in the title are collected over a period of “all years” and 3,670 papers are available (as of 16 August 2021).

### Methods

There are many Scientometric tools developed to identify and visualize hotspots, evolution, and emerging trends within a specific field, such as Citespace, VOS viewer, Bibliometrics, Sci2, SciMAT, SATI, and Bibexcel. Among these Scientometric tools, Citespace is regarded as the most popular software to perform data analysis in terms of data acquisition, data processing, visualization, and interpretation (32). First, Citespace has an advantage on the academic search of the Web of Science (WOS) core or other databases compare with Bibliometrix, Sci2, SciMAT, SATI, and Bibexcel. Second, some key functions and important metrics in CiteSpace can be used for more convenient and accurate data processing. It is beneficial to detect the research frontier in a particular field using the function of burst detection supported by Citespace to identify features with high intensity in a limited time (33). Third, a variety of visualization methods, especially in burst detection, time zone views, time distance measured by warm and cold colors make visual map a better readable and easier interpretation. Last but not the least, Citespace is widely used in Consumer Privacy Research (34), online learning (35), hospitality Research (36), intercultural competence (37), and other disciplines. Given the powerful function and advantages of Citespace, this study applies it to health literacy research.

## Results

### Visualization and Analysis on Yearly Quantitative Distribution of Scientific Output

**Figure 1:**
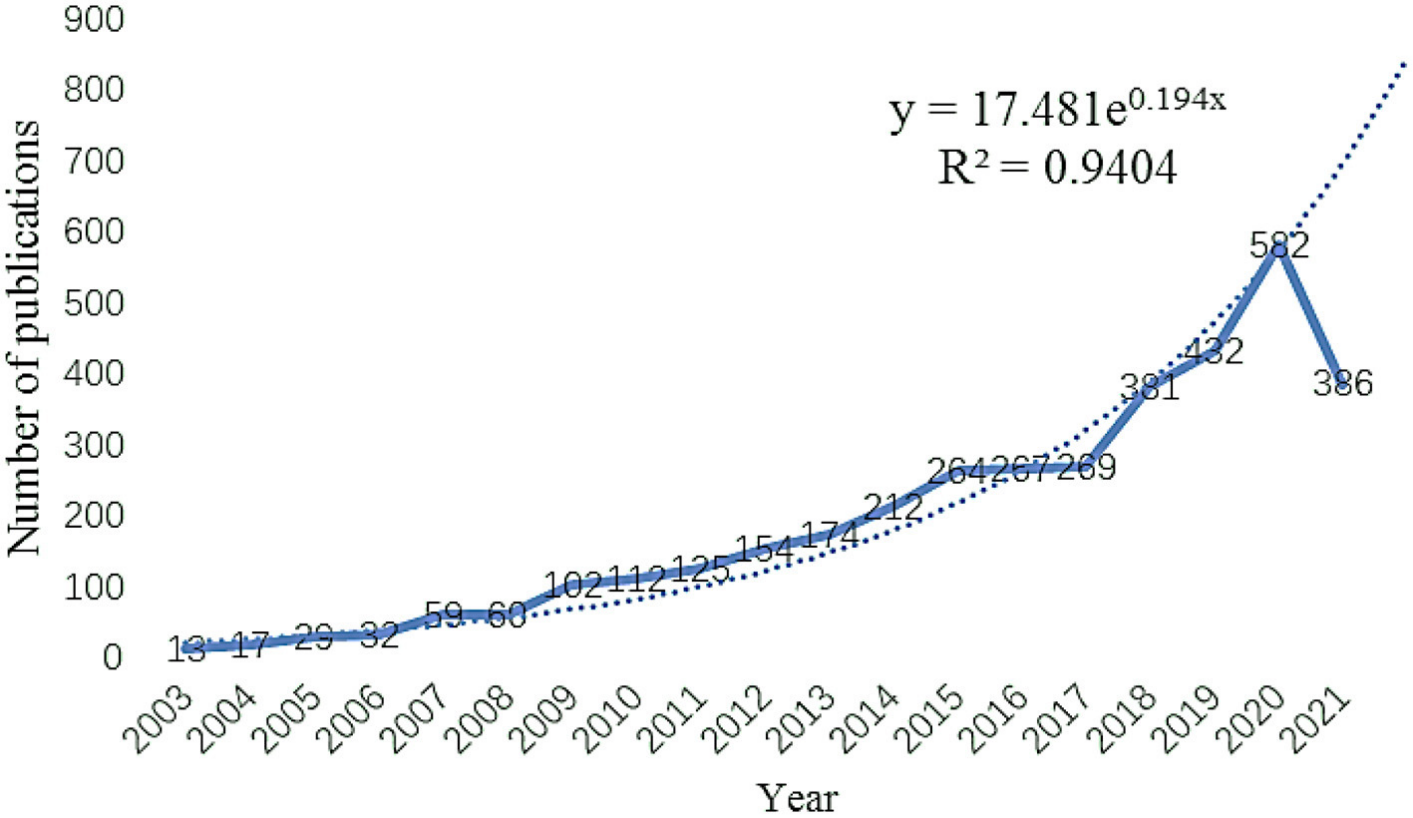
Studies selection flow diagram.

The number of published papers over time plots the annual changes in the attention paid by international experts and scholars to health literacy. Figure 1 indicates the total annual number of statistical results for 3,670 literature data related to health literacy in the WOS core collection, published from 2003 to 2021. A total of 3,670 publications on health literacy consists of articles and reviews, with an average annual publication of 193 papers. Based on the line chart of the number of annual publications, it can be seen that there is a continuous macro upward development trend and scholars' interests. In a word, the historical development of health literacy research can be split into the following four sub-periods:

Initial germination stage (2003–2008): During the first stage, the number of related published papers was much more limited. Since the first article was published in 2003 with health literacy as a title, there was a slowly increasing trend from 2003 to 2008.

Initial slow-growth period (2009–2014): In 2009, there is a sudden upward trend in publications as compared to the amount of research in the previous year and, on average all is over 100 papers.

Steady growth stage (2015–2017): 2015 to 2017 is the steady stage wherein the number of articles published each year is around 260 articles with slight fluctuations until it increased sharply again in 2018, reaching 381.

Rapid development stage (2018–2020): The number of published articles on health literacy increased rapidly from 2018 to 2020. The number of articles has reached 582 in 2020, which is nearly 45 times higher than the number of articles in 2003. The potential increase of the annual publication outputs can be forecasted according to the following fitting curve *y* = 17.462*e*^0.1941*x*^ with *R*^2^ = 0.941, as shown in Figure 1. The number of articles published in 2021 is expected to increase up to 700.

### Visualization and Analysis on Main Source Journals and Discipline Distribution

The research literature on health literacy refers to 176 journals during 2003–2021. Based on the JCR categories, these cited journals are found to involve various disciplines like Health Care Sciences and Services, Medicine, General and Internal, Public, Environmental and Occupational Health, Social Sciences, Interdisciplinary, Communication, Information Science & Library Science, Health Policy and Services, Social Sciences, Biomedical, Primary Health Care, Multidisciplinary Sciences, Pediatrics, Medical Informatics and so on.

**Figure 2:**
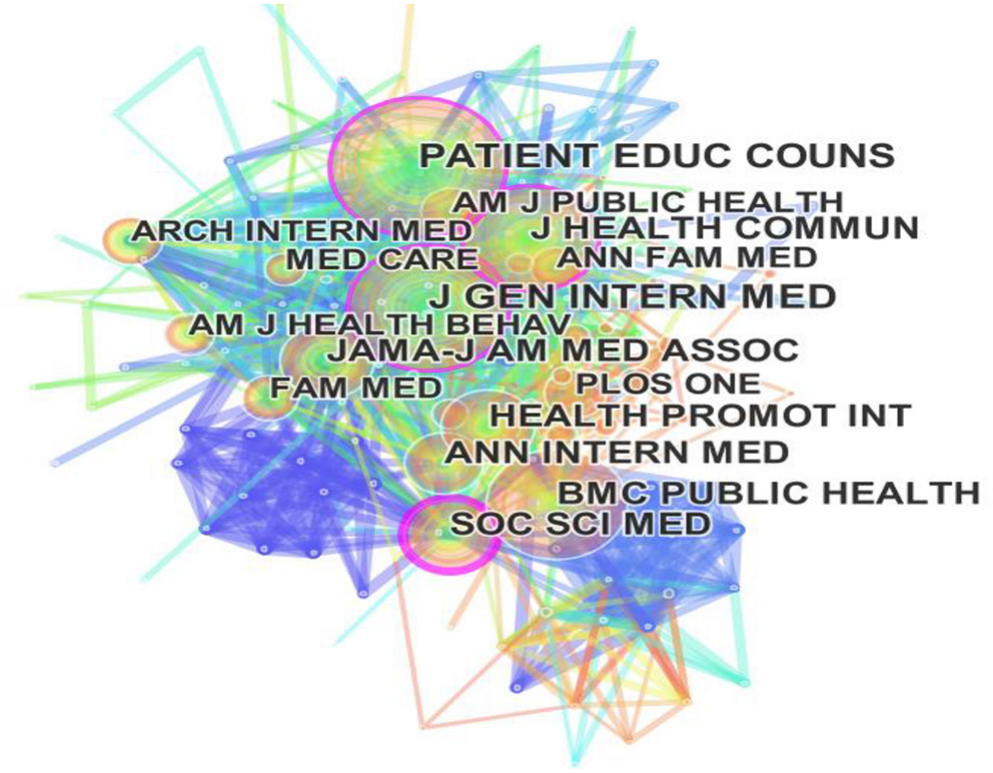
Visualization on main research journals in health literacy research.

The visualization of the journal co-citation network is plotted as shown in Figure 2; Table 1 below illustrates a list of the top 20 journals in the field of health literacy. The centrality and cited frequency of the literature published by a journal are the key indicators to evaluate the influence and importance of the core journal in a research field. The “Soc Sci Med,” “J Health Commun,” “Patient Educ Couns” and “J Gen Intern Med” play important roles in the evolution of health literacy according to a statistical analysis of centrality. The purple rings around the outer rim of some nodes of these academic journals indicate they have a closer interrelationship with other journals. From the perspective of the cited Frequency, the most highly cited journal related to health literacy research is Gen Intern Med” with the frequency of 2,042. The “Patient Educ Couns” “J Health Commun” and “BMC Public Health” in Figure 2 are three highly cited journals that corresponded with the relatively large node. Among them, the other journals with high cited frequency such as “Jama-J Am Med Assoc,” “Health Promot Int,” “Soc Sci Med” and “Ann Intern Med” are also of great value for this area. In terms of the dual criteria of impact factor and JCR category, the most influential journals publishing health literacy research are the Lancet (*IF* = 79.321, Q1), Jama-J Am Med Assoc (*IF* = 56.272, Q1), Ann Intern Med (*IF* = 25.381, Q1).

**Table 1:** The top 20 journal in the field of health literacy research.

**Rank**	**Frequency**	**Centrality**	**Journal names**	**IF in 2021**	**WoS category**	**JCR category**
1	2,042	0.12	J Gen Intern Med	5.128	HCS&S; MG&I	Q1
2	1,978	0.13	Patient Educ Couns	2.94	PE&OH; SSI	Q2; Q1
3	1,624	0.15	J Health Commun	2.781	C; IS & L S	Q2
4	1,495	0.06	BMC Public Health	3.295	PE&OH	Q2
5	1,336	0.05	Jama-J Am Med Assoc	56.272	MG&I	Q1
6	1,211	0.02	Health Promot Int	2.483	HP&S; PE&OH	Q3; Q2
7	1,143	0.35	Soc Sci Med	4.634	PE&OH; SSB	Q1
8	1,134	0.08	Ann Intern Med	25.381	MG&I	Q1
9	923	0.06	Fam Med	1.756	PHC; MG&I	Q3; Q4
10	912	0.03	Am J Public Health	9.308	PE&OH	Q1
11	887	0.05	Arch Intern Med	3.277	MG&I	Q1
12	771	0.00	PLoS ONE	3.24	MS	Q2
13	764	0.04	Ann Fam Med	5.166	PHC; MG&I	Q1
14	731	0.01	Am J Health Behav	1.97	PE&OH	Q3
15	685	0.09	Med Care	2.983	HCS&S; HP&S; PE&OH	Q2
16	564	0.15	Pediatrics	7.124	P	Q1
17	558	0.03	Lancet	79.321	MG&I	Q1
18	549	0.12	Am J Perv Med	5.043	MG&I; PE&OH	Q1
19	543	0.00	J Med Internet Res	5.428	HCS&S; MI	Q1
20	522	0.00	BMC Health Serv Res	2.655	HCS&S	Q3

*^a^Source: Web of science and journal citation reports 2021*.

*HCS&S, health care sciences & services; MG&I, medicine, general & internal; PE&OH, public, environmental & occupational health; SSI, social sciences, interdisciplinary; C, communication; IS&LS, information science & library science; HP&S, health policy & services; SSB, social sciences, biomedical; PHC, primary health care; MS, multidisciplinary sciences; P, pediatrics; MI, medical informatics*.

### Visualization and Analysis on Main Countries and Their Cooperation

**Figure 3:**
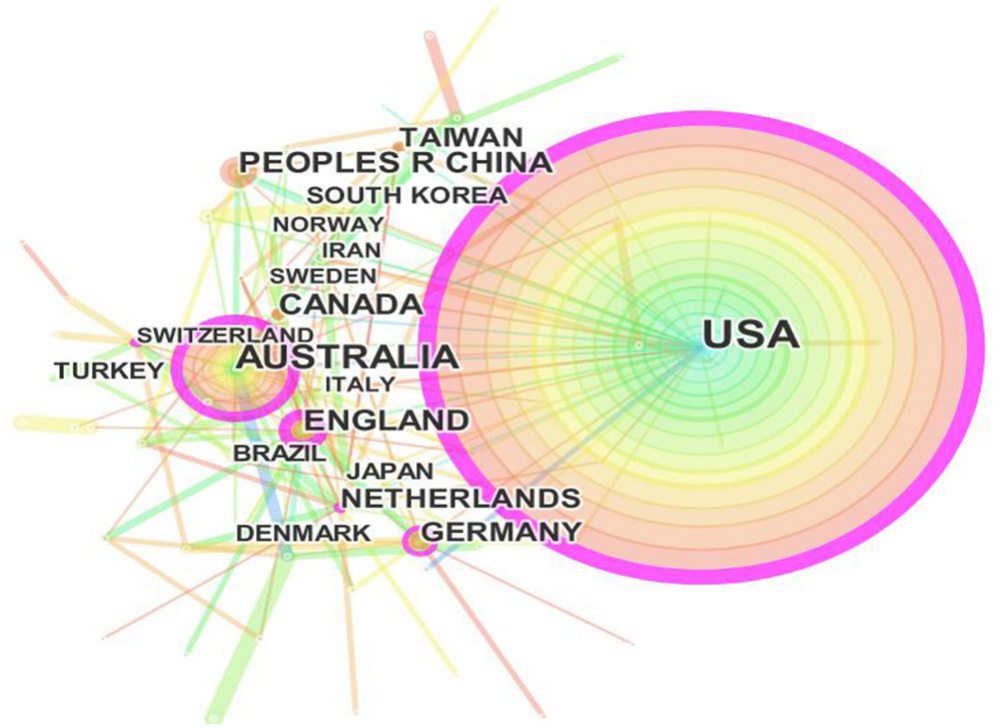
Visualization on main research countries in health literacy research.

According to the cooperation network of analysis results between various countries, the publications on health literacy research come from 87 countries and territories, and the top−10 most influential countries and territories with the largest number of published papers are shown in Figure 3; Table 2. There is no doubt that the biggest node in the USA, which indicates the USA makes the largest contributions to health literacy research and ranks first in the number of publications with the frequency of 1,618. In terms of the centrality of published papers at each node, the USA also keep first place among the degree of communication between the connected countries/regions of publications from 2003 to 2021. Therefore, the USA is the most active and influential research country in the research field of health literacy research.

**Table 2:** The top 10 country/territory in the field of health literacy research.

**Rank**	**Country/Territory**	**Religion**	**Publications**	**Centrality**
1	USA	North America	1,618	0.42
2	Australia	Oceania	409	0.32
3	People R China	Asia	180	0.01
4	Canada	North America	168	0.03
5	England	Western Europe	155	0.29
6	Germany	Central Europe	140	0.11
7	Netherlands	Western Europe	109	0.2
8	Taiwan (China)	Asia	93	0.08
9	South Korea	Asia	87	0.00
10	Japan	Asia	76	0.01

Meanwhile, Australia, People R China, Canada, England, Germany, Netherlands, Taiwan (China), South Korea, Japan, and other countries or territories make decreasing contributions to health literacy research. The number of publications in Australia has published over 400 publications, ranking second, followed by People R China, with 180 papers published. Canada, England, Germany, and Netherlands published more than 100 but <170 papers. Taiwan (China), South Korea, Japan, and other countries are <100 papers. Most of the productive research countries are from North America and Europe. In a word, there is an actual uneven geographical distribution and the cooperative relationship with other countries of the number of articles published by each country or territory.

### Visualization and Analysis on Main Institutes and Their Cooperation

**Figure 4:**
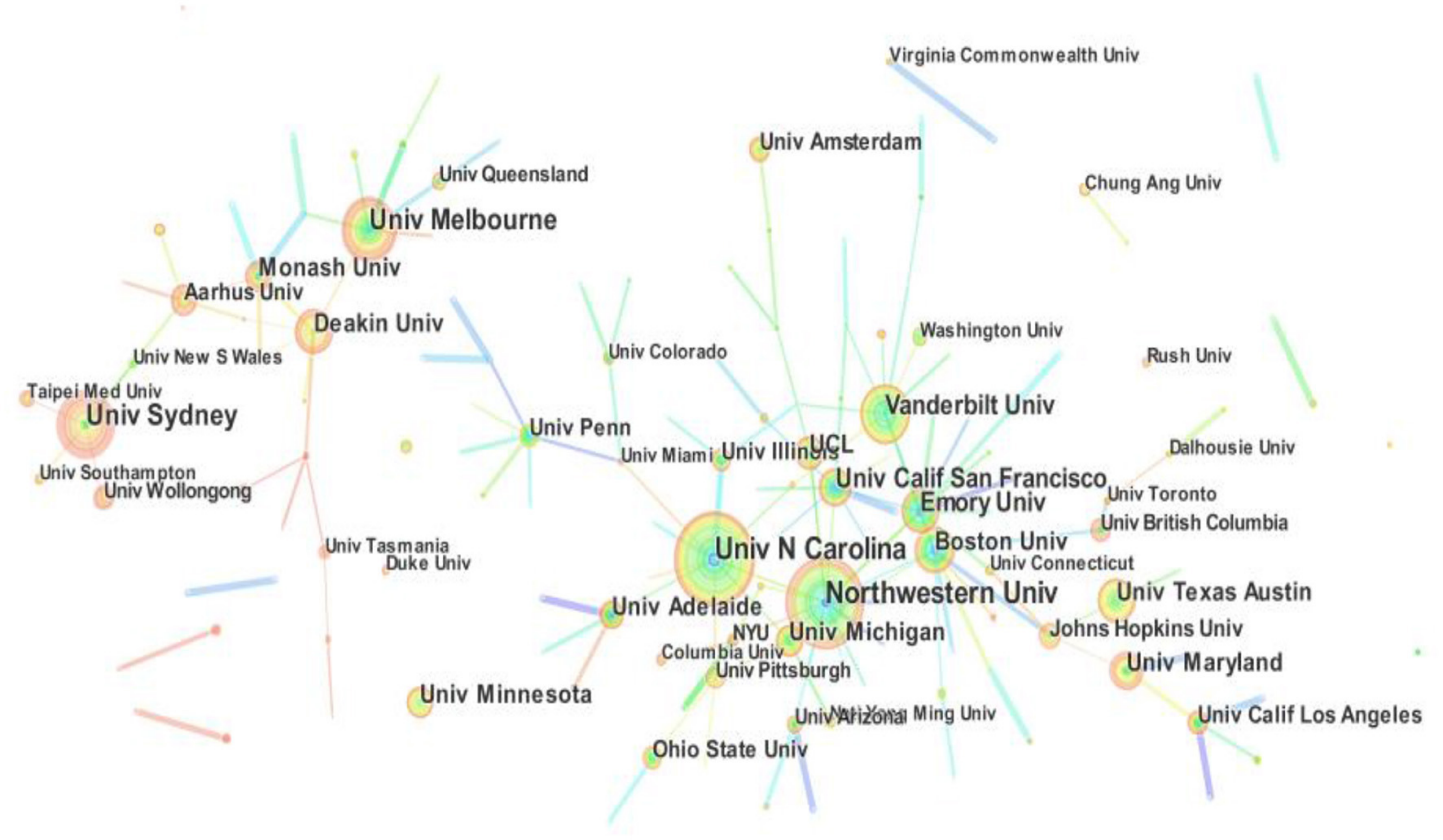
Visualization on main research institutes in health literacy research.

The leading research institutes of health literacy research are presented in Figure 4; Table 3. In Figure 4, there are 394 institutions of publications. The thickness and quantity of the ties indicate the collaboration degree among institutions. In terms of Figure 4, the relationship in the cooperation between the key institutions focuses on the cooperation between domestic institutions, such as the strong tie between Univ N Carolina and Univ Illinois, the connection of Northwestern Univ and Emory Univ and linkages in the relationship between Univ Melbourne and Deakin Univ. The thicker the connecting line, the higher degree of link strength between the different institutions, and vice versa.

**Table 3:** The top 10 institution in the field of health literacy research.

**Rank**	**Institution**	**Country / Territory**	**Publications**	**Burst**
1	Univ N Carolina	USA	72	4.40
2	Northwestern Univ	USA	70	6.53
3	Univ Melbourne	Australia	54	4.87
4	Univ Sydney	Australia	54	0.00
5	Vanderbilt Univ	USA	47	6.53
6	Univ Calif San Francisco	USA	41	5.33
7	Emory Univ	USA	41	6.96
8	Boston Univ	USA	39	5.34
9	Univ Texas Austin	USA	38	3.98
10	Deakin Univ	Australia	36	0.00

Table 3 presents the top 10 institutions in the field of health literacy research. The top-10 core academic institutions in the collaboration network graph conclude seven American institutes and three Australian institutes. In terms of the node size and publications, Univ N Carolina (72) and Northwestern Univ (70) are the largest number of academic outputs among these institutes, followed by Univ Melbourne (54), Univ Sydney (54), Vanderbilt Univ (47), Univ Calif San Francisco (41), Emory Univ (41). The remaining institutes with more than 30 and <40 papers are expected to continue outstanding contributions to the field of health literacy research, such as Boston Univ (39), Univ Texas Austin (38), and Deakin Univ (36). In addition, Emory Univ, Vanderbilt Univ, and Northwestern Univ have a burst value over 6. In terms of the burst of publications in Table 3, which means that these universities have strong power in this field over a short period.

### Visualization and Analysis on Main Contributors and Their Cooperation

**Figure 5:**
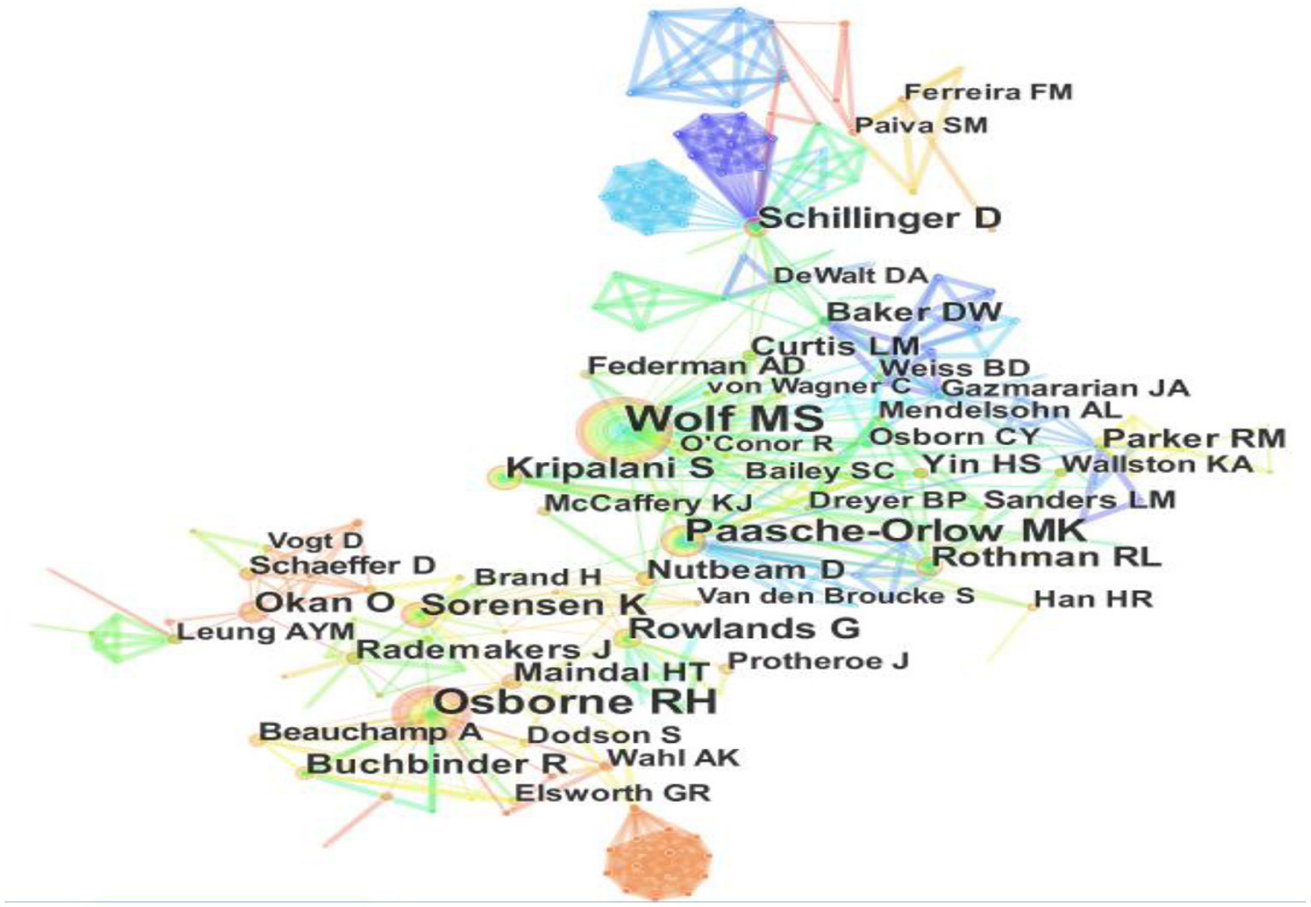
Visualization on main research contributors in health literacy.

The author collaboration networks of main contributors are plotted as Figure 5, with 699 authors as well as 1,162 collaboration ties described. Besides, the top 10 most influential authors from the output of CiteSpace are listed in Table 4.

**Table 4:** The top 3 contributors in the field of health literacy research.

**Author**	**Institutions**	**Publications**
Wolf MS	Northwestern Univ	73
Osborne RH	Deakin Univ	59
Paasche-Orlow MK	Boston Univ	43
Sorensen K	Maastricht Univ	31
Kripalani S	Emory Univ	31
Rowlands G	Aarhus Univ	27
Schulz P J	Univ Della Svizzera Italiana	27
Lee HY	Univ Minnesota	26
Schillinger D	Univ Calif San Francisco	26
Rothman RL	Vanderbilt Univ	25

Wolf MS ranks on the top of 10 highly cited scholars in the field of health literacy research. He comes from Northwestern University. His main research contributions are the relationship between health literacy and health management, health status, health outcomes, health risk behaviors, health disparities, health literacy skills, and health beliefs. The older people are the main data source collected in his health literacy research. Osborne RH, from Deakin University, ranks second. Prof. Osborne RH tends to develop health literacy scale tools, such as health literacy measure tool, health literacy assessment tool, and the health literacy management scale, to evaluate health outcomes, investigate health literacy needs, health behavior. In addition, he also pays more attention to the effect of electronic health literacy on health outcomes of socially disadvantaged groups, such as older adults, ethnic minority groups, low-income groups, low-literacy groups, and rural communities (31).

Paasche-Orlow MK, working at Boston Univ, focuses on how to improve limited health literacy to reduce health disparities. He argues that the application of information technology in health literacy can reduce disparities by using intelligent, convenient, and actionable systems which provide information, advice, and behavioral support to low-literacy populations at the place and time it is needed (32).

The other influential scholars on health literacy are Sorensen K, Kripalani S, Rowlands G, Schulz P J, Lee HY, Schillinger D, Rothman RL. These researchers make further contributions on definitions and conceptual frameworks of health literacy, Health Literacy Measurement, and health behavior, and so on.

### Visualization and Analysis on the Highly Cited Articles

The advantage of highly cited papers for bibliometric analysis is very obvious, that is, the highly cited papers are regarded as one of the most important symbols to evaluate the influence of the general development trend and research frontiers (33). Based on the data, 3,674 papers, this study use Price's law to identify highly cited papers. M is computed to identify the lowest cited frequency of highly cited papers. The formula is as follows:


$$\begin{array}{l} m=0.749\sqrt{n_{\text{max}}} \end{array}$$

Where *n*_*max*_ is citation frequency of highly cited papers.

By employing the formula, publications that were cited beyond 32 times were generally referred to as highly cited. Finally, a total of 637 highly cited articles is available.

**Figure 6:**
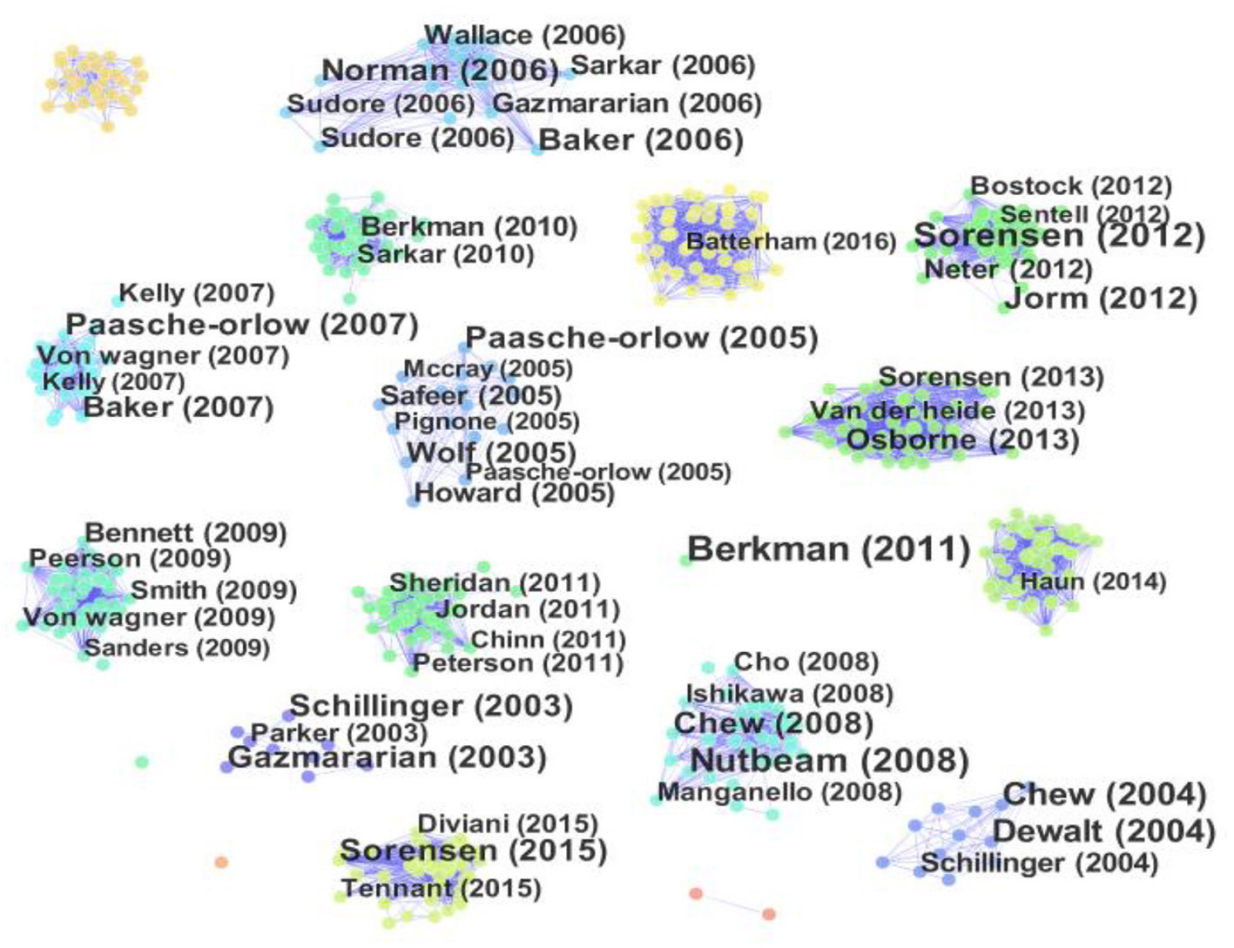
Visualization on highly cited papers articles in health literacy research.

The top 10 highly cited articles, including title, year, authors, journal, and citation, are shown in Figure 6; Table 5. Based on the abstract of these articles, the scope of these most influential papers in the field of health literacy focuses on low health literacy, health outcomes, the concept of health literacy, and eHealth literacy. Specifically, 3 out of 10 articles are review articles. The top 2 highly cited papers is published in 2011 and 2012, respectively. Other articles were published during 2003–2008. It is also observed that most articles were co-authored. The top 10 highly cited articles were published in seven different journals, among which four papers were from J GEN INTERN MED, and one each from ANN INTERN MED, BMC PUBLIC HEALTH, SOC SCI MED, FAM MED, ARCH INTERN MED, and J MED INTERNET RES. The article titled “Low Health Literacy and Health Outcomes: An Updated Systematic Review” has the highest citation frequency with 1,824 and suggests it to be the most important article. The number of another three articles also has been cited over 1,000 times, which highlights its importance in the field of health literacy.

**Table 5:** The top 10 papers with the most citations.

**Title**	**Year**	**Authors**	**Journal**	**Citation**
Low Health Literacy and Health Outcomes: An Updated Systematic Review	2011	Berkman ND, Sheridan SL, Donahue KE	ANN INTERN MED	1,824
Health literacy and public health: A systematic review and integration of definitions and models	2012	Sorensen K, Broucke S, Fullam J, et al.	BMC PUBLIC HEALTH	1,492
The evolving concept of health literacy: New directions for health literacy studies	2008	Nutbeam, D	SOC SCI MED	1,052
Literacy and health outcomes - A systematic review of the literature	2004	Dewalt DA, Berkman ND, Sheridan S, et al.	J GEN INTERN MED	1,019
Brief questions to identify patients with inadequate health literacy	2004	Chew, LD; Bradley, K A and Boyko, EJ	FAM MED	984
Closing the loop - Physician communication with diabetic patients who have low health literacy	2003	Schillinger D, Piette J, Grumbach K, et al.	ARCH INTERN MED	735
Validation of screening questions for limited health literacy in a large VA outpatient population	2008	Chew L, Griffin J M, Partin MR, et al.	J GEN INTERN MED	729
eHealth literacy: Essential skills for consumer health in a networked world	2006	Norman CD, Skinner HA	J MED INTERNET RES	722
The prevalence of limited health literacy	2005	Paasche-Orlow MK Parker RM, Gazmararian JA, et al.	J GEN INTERN MED	691
The meaning and the measure of health literacy	2006	Baker DW	J GEN INTERN MED	605

### Visualization and Analysis on Keywords Co-occurrence

**Figure 7:**
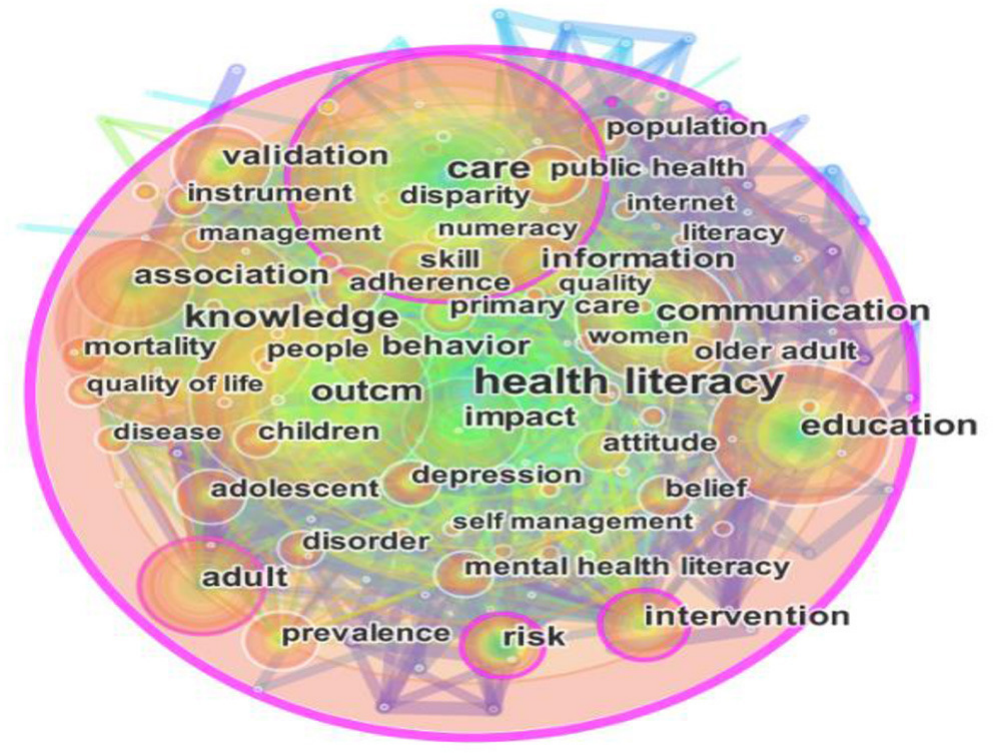
Visualization on co-occurring keywords network.

The core and focus of research topics in a specific knowledge domain can be generalized by keywords. The keywords co-occurrence of health literacy are clearly illustrated in Figure 7 with Citespace and the top 20 keywords with high Frequency, centrality, and burst strength in health literacy are presented in Table 6. Every node in Figure 7 represents a keyword. The frequency occurrence of keywords can be measured by the size of the node, which is an important indicator to judge the importance of keywords. Otherwise, the keyword with a high centrality also is a symbol of the current research hotspots and emerging research trends. According to Figure 7; Table 6, the node of “health literacy” ranks first of all the keywords with the highest count of 1,955 and centrality of 0.23. Regardless of the keyword “health literacy,” the other keywords such as “Care,” “knowledge,” “Outcm (Outcome),” “Education,” “Communication,” “Adult,” “Impact,” “Association” and “Behavior” at the Frequency range from 745 to 293. Further, the keywords such as “Risk,” “Intervention.” “Breast cancer,” “Care,” “Adult,” “knowledge,” “Education,” “Association” and “Behavior” have a larger centrality compared with others, which indicated that they play a more active role in connectivity in the keywords network.

**Table 6:** The top 20 keywords with high frequency, centrality and burst strength in health literacy.

**No**.	**Frequency**	**Keywords**	**Centrality**	**Keywords**	**Burst strength**	**Keywords**
1	1,955	Health literacy	0.23	Health literacy	22.5	Literacy
2	745	Care	0.12	Risk	13.27	Medicare enrollee
3	673	knowledge	0.12	Intervention	13.04	Knowledge
4	620	Outcm	0.12	Breast cancer	12.06	Hospital admission
5	472	Education	0.11	Care	11.25	Readability
6	429	Communication	0.11	Adult	9.31	Reading ability
7	356	Adult	0.09	knowledge	8.06	Chronic disease
8	329	Impact	0.07	Education	7.12	Managed care enrollee
9	323	Association	0.07	Association	7.08	Enrollee
10	293	Behavior	0.06	Behavior	6.72	Risk

Meanwhile, this study can judge the popularity of certain keywords in a short time according to burst terms. It could be seen that “literacy,” “Medicare enrollee,” “knowledge,” “hospital admission” and “readability” were the top five research frontiers of health literacy as shown in Table 6. Based on burst strength, the research frontiers of health literacy research can be concluded into the following three categories. First of all, a functional level of health literacy offered by Nutbeam remains a research hotspot (34). Reading ability is an important component of all basic skills of literacy. Beker et al. argued that there is a higher risk of hospital admission for individuals with inadequate or marginal functional health literacy (35). Secondly, data sources for many papers were mainly from Medicare enrollees or managed care enrollees aged 65 or older. Elderly individuals have worse knowledge and self-management skills which is suitable to investigate the relationship between low health literacy and health outcomes (36). Thirdly, the elder with a higher prevalence of chronic diseases, such as asthma, diabetes, congestive heart failure, hypertension, has a heavy burden of medical knowledge due to frequent use of health care services (37).

**Figure 8:**
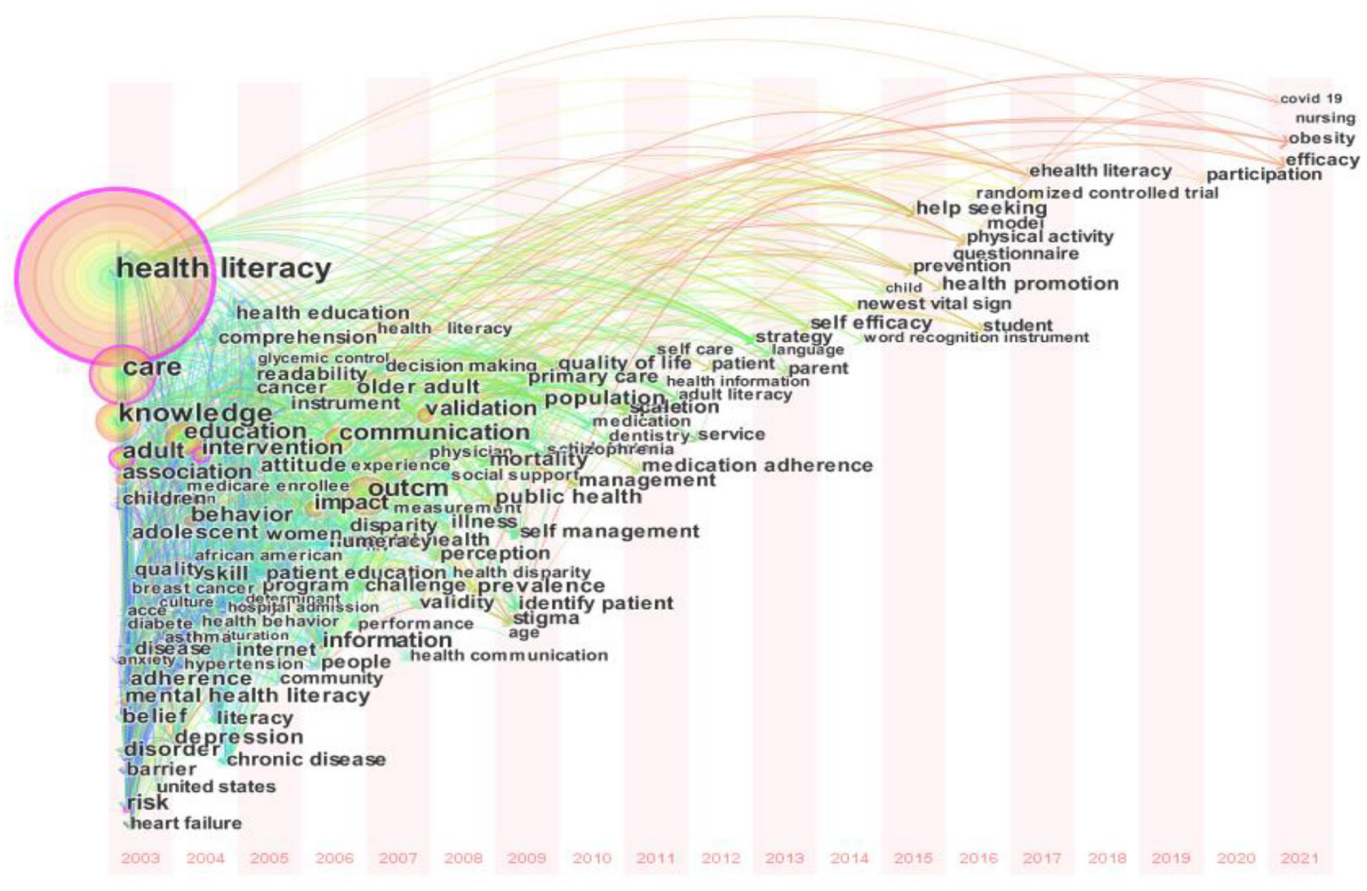
Time-zone map of co-occurring keywords network on health literacy research.

In addition, the time zone map shows the evolution of the prevalent frontiers of the core research topic over an approximately 20-year period as shown in Figure 8. In the Initial stage (from 2003 to 2010), diversified development in the field of health literacy is emerging and a lot of high-frequency keywords appeared, such as health literacy, care, knowledge, outcome, and education et al. Over the past 2011–2016 years, the main research hotspots in health literacy research are centered on medication adherence, strategy, self-efficacy, health promotion, prevention and help seeking et al. For the most recent 5 years (from 2017 and 2021), some recent studies take some new topics into accounts, such as eHealth literacy, COVID-19, nursing, and obesity.

## Discussion

### Main Finding

This study conducts a visualized and Scientometric analysis of health literacy research and the main findings are discussed in detail as follows:

First, this study has been a scientific review of 3,670 publications and shows a significantly strong increasing trend of health literacy research during the period of 2003 to 2020. This is consistent with the prior result (30). In addition, this study forecast the publication amount will reach a higher level in 2021 because of the more extensive attention in the academic field.

Regarding the core journals, journal co-citation analysis identified the top 20 influential journals in the health literacy domain. It is clear that health literacy research attracts more and more attention because high-quality journals, such as Lancet (*IF* = 79.321, Q1), Jama-J Am Med Assoc (*IF* = 56.272, Q1), Ann Intern Med (*IF* = 25.381, Q1), et al., have a significant impact on diffusion scope.

As far as the main strength of health literacy research was concerned, the two most outstanding countries are USA and Australia in this area. Based on the publication amount of each institute, the top 10 institutions in the field of health literacy research all come from USA and Australia. In addition, better cross-national co-operations should be strengthened in the research process compared with strong cooperation between domestic institutions, according to the result of visualized and Scientometric analysis.

Through in-depth analysis of contributors, individual scholars with the most published articles are Wolf MS from Northwestern University, Osborne RH from Deakin University, and Paasche-Orlow MK from Boston Univ, who made significant contributions in this field.

Our results showed that the highly cited articles highlighted and synthesized some important issues of theoretical knowledge and empirical studies in the field of health literacy, including the relationship between low health literacy and health outcomes, the evolving concept of health literacy, the measurement of health literacy as well as patients' health literacy skills on accessing and effectively applying information technology (38).

Apart from the above discovery, this study use three indicators, namely keyword frequency, keyword centrality, and keyword burst, to investigate the research hotspots of health literacy. Some high-frequency keywords, such as health literacy, care, knowledge, outcome and education, can better show the current focus of the theme of change and evolution. The high-centrality keywords indicate the related terms, involving health literacy, risk, intervention, breast cancer, and care et al., are at the core of health literacy research. Based on the statistics of keywords with high bursts, this research find that kinds of enrollee, literacy, knowledge, and chronic disease are important directions in health literacy research. Linking this result with previous literature, a possible explanation is that Medicare managed care enrollees are mainly 65 or older with inadequate or marginal health literacy and chronic disease, who have urgent demand to increase health knowledge (35, 39).

Last but not least, many scholars pay more attention to health literacy (12, 40–42), COVID-19 (6, 16, 17, 42–44), nursing (12, 45) and obesity (46, 47), just as time zone map has shown. For example, some scholars find that whether information technology in health care has the potential to improve the self-management level of patients with chronic diseases is associated with intended users' literacy (48, 49). The massive open online courses (MOOCs) are beneficial to promote digital health literacy (50). During the COVID-19 pandemic, higher health literacy and eHealth literacy is beneficial for the public to prevent disease diffusion and promote both personal and organizational security (51). For the nursing setting, low health literacy harms health outcomes and the costs of the healthcare system (52). Many studies investigate the relationship between health literacy and obesity, arguing that populations with higher health literacy can increase people's control over their weight (46).

### Limitation and Future Research

Inevitably, a few inherent limitations of this visualized and Scientometric study are listed as follows. First, the datasets this research use were published in English and other articles are excluded for reasons such as the usage of Korean. Therefore, this may affect the results of the analysis to some extent due to the lack of top journals from non-English speaking countries and the limit by the given data sources. Second, this study may underestimate the influence of the newly published articles. In future research, how to use other analysis tools is important to discover the potential value of the newly published articles. Third, this study only integrates the data of Web of Science and ignores the data of PubMed. Because PubMed data is open access, it may be more advantageous to collect PubMed data and may highlight different pictures. Therefore, it is recommended that future scholars collect data from various other sources, especially PubMed, to further analyze this concept in more detail.

## Data Availability Statement

The original contributions presented in the study are included in the article/supplementary material, further inquiries can be directed to the corresponding author.

## Author Contributions

JW: concept, data collection, data analysis, writing, and revising the manuscript. FS: data analysis, writing, reviewing and editing the manuscript.

## Funding

This research was funded by the Key Program of Teaching Reform in Higher Education of Heilongjiang Province, grant number SJGZ20200148 and Henan Education Department of Humanities and Social Sciences Research Project Grant Number: 2022-ZZJH-420.

## Conflict of Interest

The authors declare that the research was conducted in the absence of any commercial or financial relationships that could be construed as a potential conflict of interest.

## Publisher's Note

All claims expressed in this article are solely those of the authors and do not necessarily represent those of their affiliated organizations, or those of the publisher, the editors and the reviewers. Any product that may be evaluated in this article, or claim that may be made by its manufacturer, is not guaranteed or endorsed by the publisher.

## References

[1] Callşkan C Üner S. Disaster literacy and public health: a systematic review and integration of definitions and models. Disaster Med Public Health Prep. (2020) 18:1–10. 10.1017/dmp.2020.100 32618555

[2] Sørensen K Pelikan JM Röthlin F Ganahl K Slonska Z Doyle G. Health literacy in Europe: comparative results of the European health literacy survey (HLS-EU). Eur J Public Health. (2015) 25:1053–8. 10.1093/eurpub/ckv043 25843827 PMC4668324

[3] Paasche-Orlow MK Parker RM Gazmararian JA Nielsen-Bohlman LT Rudd RR. The prevalence of limited health literacy. J Gen Intern Med. (2005) 20:175–84. 10.1111/j.1525-1497.2005.40245.x 15836552 PMC1490053

[4] Choudhry FR Ming LC Munawar K Zaidi STR Patel RP Khan TM. Health literacy studies conducted in Australia: a scoping review. Int J Environ Res Public Health. (2019) 16:1–32. 10.3390/ijerph16071112 30925706 PMC6479782

[5] Finlay S Meggetto E Robinson A Davis C. Health literacy education for rural health professionals: shifting perspectives. Aust Heal Rev. (2019) 43:404–7. 10.1071/AH18019 30032741

[6] Patil U Kostareva U Hadley M Manganello JA Okan O Dadaczynski K. Health literacy, digital health literacy, and COVID-19 pandemic attitudes and behaviors in US college students: Implications for interventions. Int J Environ Res Public Health. (2021) 18:1–14. 10.3390/ijerph18063301 33806763 PMC8004744

[7] Rowlands G Protheroe J Winkley J Richardson M Seed PT Rudd R. Mismatch between population health literacy and the complexity of health information: an observational study. Br J Gen Pract. (2015) 65:379–86. 10.3399/bjgp15X685285 26009533 PMC4439828

[8] Nutbeam D. The evolving concept of health literacy. Soc Sci Med. (2008) 67:2072–8. 10.1016/j.socscimed.2008.09.050 18952344

[9] Mansfield E Wahba R De Grandpré E. Integrating a health literacy lens into nutrition labelling policy in Canada. Int J Environ Res Public Health. (2020) 17:1–7. 10.3390/ijerph17114130 32531887 PMC7312128

[10] Kosiyaporn H Julchoo S Sinam P Phaiyarom M Kunpeuk W Pudpong N. Health literacy and its related determinants in migrant health workers and migrant health volunteers: a case study of Thailand, 2019. Int J Environ Res Public Health. (2020) 17:2105. 10.3390/ijerph17062105 32235732 PMC7143383

[11] Li C Guo Y. The effect of socio-economic status on health information literacy among urban older adults: evidence from western China. Int J Environ Res Public Health. (2021) 18:3501. 10.3390/ijerph18073501 33800562 PMC8036692

[12] Kim S Oh J. The relationship between e-health literacy and health-promoting behaviors in nursing students: A multiple mediation model. Int J Environ Res Public Health. (2021) 18:5804. 10.3390/ijerph18115804 34071469 PMC8199246

[13] Juvinyà-Canal D Suñer-Soler R Porquet AB Vernay M Blanchard H Bertran-Noguer C. Health literacy among health and social care university students. Int J Environ Res Public Health. (2020) 17:1–10. 10.3390/ijerph17072273 32230985 PMC7177671

[14] Levin-Zamir D Bertschi I. Media health literacy, Ehealth literacy, and the role of the social environment in context. Int J Environ Res Public Health. (2018) 15:1–12. 10.3390/ijerph15081643 30081465 PMC6121358

[15] Choi M. Association of ehealth use, literacy, informational social support, and health-promoting behaviors: Mediation of health self-efficacy. Int J Environ Res Public Health. (2020) 17:1–12. 10.3390/ijerph17217890 33126469 PMC7662976

[16] Zakar R Iqbal S Zakar MZ Fischer F. COVID-19 and health information seeking behavior: digital health literacy survey amongst university students in Pakistan. Int J Environ Res Public Health. (2021) 18:4009. 10.3390/ijerph18084009 33920404 PMC8069684

[17] Sentell T Vamos S Okan O. Interdisciplinary perspectives on health literacy research around the world: more important than ever in a time of covid-19. Int J Environ Res Public Health. (2020) 17:1–13. 10.3390/ijerph17093010 32357457 PMC7246523

[18] Kim MY Oh S. Nurses' perspectives on health education and health literacy of older patients. Int J Environ Res Public Health. (2020) 17:1–11. 10.3390/ijerph17186455 32899759 PMC7558471

[19] Lee HE Lori JR. Scoping review: valid and reliable health literacy assessment tools used in low- and middle-income countries. J Nurs Meas. (2020) 28:5–22. 10.1891/JNM-D-18-00071 32179714

[20] Guzys D Kenny A Dickson-Swift V Threlkeld G. A critical review of population health literacy assessment. BMC Public Health. (2015) 15:215. 10.1186/s12889-015-1551-6 25885742 PMC4351936

[21] Warring CD Pinkney JR Delvo-Favre ED Rener MR Lyon JA Jax B. Implementation of a routine health literacy assessment at an academic medical center. J Healthc Qual. (2018) 40:247–55. 10.1097/JHQ.0000000000000116 29166290 PMC6521688

[22] Fleary SA Joseph P Pappagianopoulos JE. Adolescent health literacy and health behaviors: a systematic review. J Adolesc. (2018) 62:116–27. 10.1016/j.adolescence.2017.11.010 29179126

[23] Abdullah A May LS Salim HS Jenn NC Chinna K. Health literacy research in Malaysia: a scoping review. Sains Malaysiana. (2020) 49:1021–36. 10.17576/jsm-2020-4905-0734852001

[24] Corrarino JE. Health literacy and women's health: challenges and opportunities. J Midwifery Women's Heal. (2013) 58:257–64. 10.1111/jmwh.12018 23631442

[25] Tonsing KN. A review of mental health literacy in Singapore. Soc Work Health Care. (2018) 57:27–47. 10.1080/00981389.2017.1383335 28976296

[26] Sansom-Daly UM Lin M Robertson EG Wakefield CE McGill BC Girgis A. Health literacy in adolescents and young adults: an updated review. J Adolesc Young Adult Oncol. (2016) 5:106–18. 10.1089/jayao.2015.0059 26859721

[27] Chesser AK Keene Woods N Smothers K Rogers N. Health literacy and older adults. Gerontol Geriatr Med. (2016) 27:947–60. 10.1177/2333721416630492 28138488 PMC5119904

[28] LindlyOCrossmanMEavesMPhilpottsLKuhlthauK. Health literacy and health outcomes among children with developmental disabilities: a systematic review. Am J Intellect Dev Disabil. (2020) 125:389–407. 10.1352/1944-7558-125.5.38932936893

[29] Oliffe JL Rossnagel E Kelly MT Bottorff JL Seaton C Darroch F. Men's health literacy: a review and recommendations. Health Promot Int. (2020) 35:1037–51. 10.1093/heapro/daz077 31557281 PMC7585483

[30] Bankson HL. Health literacy: an exploratory bibliometric analysis, 1997-2007. J Med Libr Assoc. (2009) 97:148–50. 10.3163/1536-5050.97.2.016 19404510 PMC2670206

[31] Cheng C Beauchamp A Elsworth GR Osborne RH. Applying the electronic health literacy lens: systematic review of electronic health interventions targeted at socially disadvantaged groups. J Med Internet Res. (2020) 22:e18476. 10.2196/18476 32788144 PMC7453328

[32] Bickmore TW Paasche-Orlow MK. The role of information technology in health literacy research. J Health Commun. (2012) 17:23–9. 10.1080/10810730.2012.712626 23030559

[33] Liu H Chen H Hong R Liu H You W. Mapping knowledge structure and research trends of emergency evacuation studies. Saf Sci. (2020) 121:348–61. 10.1016/j.ssci.2019.09.020

[34] Nutbeam D. Health literacy as a public health goal: a challenge for contemporary health education and communication strategies into the 21st century. Health Promot Int. (2000) 15:259–67. 10.1093/heapro/15.3.259

[35] Baker DW Gazmararian JA Williams M V Scott T Parker RM Green D. Functional health literacy and the risk of hospital admission among Medicare managed care enrollees. Am J Public Health. (2002) 92:1278–83. 10.2105/AJPH.92.8.1278 12144984 PMC1447230

[36] Findley A. Low health literacy and older adults: meanings, problems, and recommendations for social work. Soc Work Health Care. (2015) 54:65–81. 10.1080/00981389.2014.966882 25588097

[37] Gazmararian JA Williams M V Peel J Baker DW. Health literacy and knowledge of chronic disease. Patient Educ Couns. (2003) 51:267–75. 10.1016/S0738-3991(02)00239-2 14630383

[38] Collins SA Currie LM Bakken S Vawdrey DK Stone PW. Health literacy screening instruments for eHealth applications: a systematic review. J Biomed Inform. (2012) 45:598–607. 10.1016/j.jbi.2012.04.001 22521719 PMC3371171

[39] Waterworth S Honey M. On-line health seeking activity of older adults: an integrative review of the literature. Geriatr Nurs (Minneap). (2018) 39:310–7. 10.1016/j.gerinurse.2017.10.016 29198622

[40] Pourrazavi S Kouzekanani K Bazargan-Hejazi S Shaghaghi A Hashemiparast M Fathifar Z. Theory-based E-health literacy interventions in older adults: a systematic review. Arch Public Heal. (2020) 78:1–8. 10.1186/s13690-020-00455-6 32793345 PMC7418312

[41] da Fonseca MH Kovaleski F Picinin CT Pedroso B Rubbo P. E-health practices and technologies: a systematic review from 2014 to 2019. Healthc. (2021) 9:1–32. 10.3390/healthcare9091192 34574966 PMC8470487

[42] Hong KJ Park NL Heo SY Jung SH Lee YB Hwang JH. Effect of E-health literacy on COVID-19 infection-preventive behaviors of undergraduate students majoring in healthcare. Healthc. (2021) 9:573. 10.3390/healthcare9050573 34066120 PMC8151528

[43] Triana AJ Gusdorf RE Shah KP Horst SN. Technology literacy as a barrier to telehealth during COVID-19. Telemed e-Health. (2020) 26:1118–9. 10.1089/tmj.2020.0155 32429770

[44] Duplaga M Grysztar M. The association between future anxiety, health literacy and the perception of the covid-19 pandemic: a cross-sectional study. Healthc. (2021) 9:43. 10.3390/healthcare9010043 33466487 PMC7824811

[45] Kim SH. Health literacy and functional health status in Korean older adults. J Clin Nurs. (2009) 18:2337–43. 10.1111/j.1365-2702.2008.02739.x 19583664

[46] Shih SF Liu CH Liao LL Osborne RH. Health literacy and the determinants of obesity: a population-based survey of sixth grade school children in Taiwan. BMC Public Health. (2016) 16:280. 10.1186/s12889-016-2879-2 27000035 PMC4802836

[47] White RO Thompson JR Rothman RL McDougald Scott AM Heerman WJ Sommer EC. Health literate approach to the prevention of childhood overweight and obesity. Patient Educ Couns. (2013) 93:612–8. 10.1016/j.pec.2013.08.010 24001660 PMC3904952

[48] Curtis LM Kwasny MJ Opsasnick L O'Conor RM Yoshino-Benavente J Eifler M. Change in health literacy over a decade in a prospective cohort of community-dwelling older adults. J Gen Intern Med. (2021) 36:916–22. 10.1007/s11606-020-06423-8 33559068 PMC8042084

[49] Norman CD Skinner HA. eHealth literacy: essential skills for consumer health in a networked world. J Med Internet Res. (2006) 8:e9. 10.2196/jmir.8.2.e9 16867972 PMC1550701

[50] Perestelo-Perez L Torres-Castaño A Gonz C Broucke SVD Gonzalo D Piccini B. IC-health project : development of MOOCs to promote digital health literacy : first results and future challenges. Sustainability. (2020) 12:6642. 10.3390/su12166642

[51] Li S Cui G Kaminga AC Cheng S Xu H. Associations between health literacy, ehealth literacy, and covid-19-related health behaviors among chinese college students: cross-sectional online study. J Med Internet Res. (2021) 23:e25600. 10.2196/25600 33822734 PMC8104003

[52] Thilo F Sommerhalder K Hahn S. Health literacy - a concept for professional nursing? Pflege. (2012) 25:427–38. 10.1024/1012-5302/a000245 23188753

